# Single-Dose Intraoperative Perampanel Infusion During Awake Glioma Surgery for Potential Prophylaxis of Intraoperative and Early Postoperative Seizures: A Case Report and Literature Review

**DOI:** 10.7759/cureus.90430

**Published:** 2025-08-18

**Authors:** Goichiro Tamura, Narushi Sugii, Koji Hirata, Masahide Matsuda, Eiichi Ishikawa

**Affiliations:** 1 Department of Neurosurgery, Mito Saiseikai General Hospital, Mito, JPN; 2 Department of Neurosurgery, University of Tsukuba Hospital, Tsukuba, JPN; 3 Department of Neurosurgery, Institute of Medicine, University of Tsukuba, Tsukuba, JPN

**Keywords:** antiseizure medication, asleep–awake–asleep, awake craniotomy, awake surgery, early postoperative seizure, insular glioma, intraoperative seizure, intravenous, perampanel, seizure prophylaxis

## Abstract

Intraoperative and early postoperative seizures are among the most critical complications associated with awake craniotomy for diffuse glioma resection. Although current guidelines do not routinely recommend prophylactic antiseizure medications (ASMs) for awake craniotomy, they are frequently used in clinical practice. However, the optimal choice of ASM remains unclear, particularly for seizure-naïve patients. Perampanel, a selective and non-competitive antagonist of α-amino-3-hydroxy-5-methyl-4-isoxazolepropionic acid-type glutamate receptors, has recently become available in an intravenous formulation. Its potential role as an intraoperative ASM during awake glioma surgery has not been previously explored. In this report, we present the first documented case of single-dose intravenous perampanel administration for potential seizure prophylaxis in awake glioma surgery. The patient was a 35-year-old woman with a left insular diffuse glioma who underwent awake craniotomy using the asleep-awake-asleep technique. A 6 mg intravenous dose of perampanel was administered at the beginning of surgery. She was awakened approximately 3.5 hours post-administration and completed motor and language tasks without difficulty. The awake phase lasted approximately 4.5 hours, during which no clinical or electrographic seizures occurred. No adverse effects, including dizziness or somnolence, were observed. Serum perampanel concentrations increased within 1-3 hours and remained elevated for one week after a single infusion (110-200 ng/mL). The concentration observed in this case was lower than the previously reported therapeutic range (200-600 ng/mL). This case represents the first reported instance of intravenous perampanel administered intraoperatively during awake glioma surgery, without significant adverse effects either during the procedure or in the early postoperative period. Its rapid therapeutic onset and sustained efficacy suggest potential utility for seizure prophylaxis in awake glioma surgery. However, further studies with larger patient cohorts are warranted to validate efficacy and establish optimal dosing strategies.

## Introduction

Resection of diffuse glioma located within or adjacent to eloquent brain areas carries a substantial risk of neurological complications, including motor, sensory, or language deficits. Awake craniotomy has emerged as an effective technique to maximize tumor resection while minimizing these risks. The most frequent causes of failed awake craniotomy are intraoperative seizures and impaired communication with patients, which may result from dysphasia, profound somnolence, or restlessness [1]. Intraoperative seizures are associated with significantly reduced likelihood of achieving gross total resection, transient or permanent neurological deficits, and increased risk of prolonged hospitalization [1-3]. The reported incidence of these seizures during awake craniotomy ranges from 2 to 20% [1-3].

The use of antiseizure medications (ASMs) for the prevention of intraoperative seizures in seizure-naïve patients undergoing awake craniotomy remains controversial. A preoperative history of seizure is a well-established risk factor for both intraoperative and early postoperative seizures [1, 4]. Current guidelines for awake craniotomy do not support the routine administration of ASMs, as previous studies have found no statistically significant difference in the incidence of intraoperative seizures between institutions that use prophylactic ASMs and those that do not [2, 5]. Additionally, concerns persist regarding potential side effects of intraoperative ASM during awake craniotomy, which may include somnolence (5-30%), confusion, impatience, agitation, or aggression (< 1%), psychotic symptoms and increased intracranial pressure (0.5-3% with phenytoin), and anxiety and irritability (1-3% with levetiracetam) [2]. Therefore, the intraoperative use of ASMs in seizure-naïve patients warrants careful consideration, based on a comprehensive risk-benefit assessment. Currently, perioperative ASMs are reportedly administered by 60-80% of U.S. neurosurgeons in glioma surgeries to mitigate the risk of early postoperative seizures [6]. However, there remains no established consensus regarding the most effective agent, appropriate dosage, or optimal timing of prophylactic ASM administration in the context of awake craniotomy.

Perampanel hydrate, a third-generation ASM, exerts its antiepileptic effects by selectively and non-competitively antagonizing α-amino-3-hydroxy-5-methyl-4-isoxazolepropionic acid (AMPA)-type glutamate receptors on the postsynaptic membrane [7]. Inhibiting AMPA-mediated excitatory neurotransmission helps suppress both focal and generalized seizure activity [4, 8]. An intravenous formulation of perampanel became available in Japan in April 2024 - preceding its release in other countries [9]. We hypothesize that intravenous perampanel may be effective in preventing seizures during awake craniotomy and in the immediately postoperative period. However, its safety and efficacy in this specific clinical setting have not yet been evaluated. We are currently conducting a prospective study to evaluate the prophylactic use of perampanel in this context, approved by the Tsukuba University Clinical Research Review Board (approval number: TCRB23-026). Written informed consent was obtained from the patient. Here, we present the first documented case of awake craniotomy in which intravenous perampanel was administered with the intent to prevent intraoperative and early postoperative seizures.

## Case presentation

**Figure 1:**
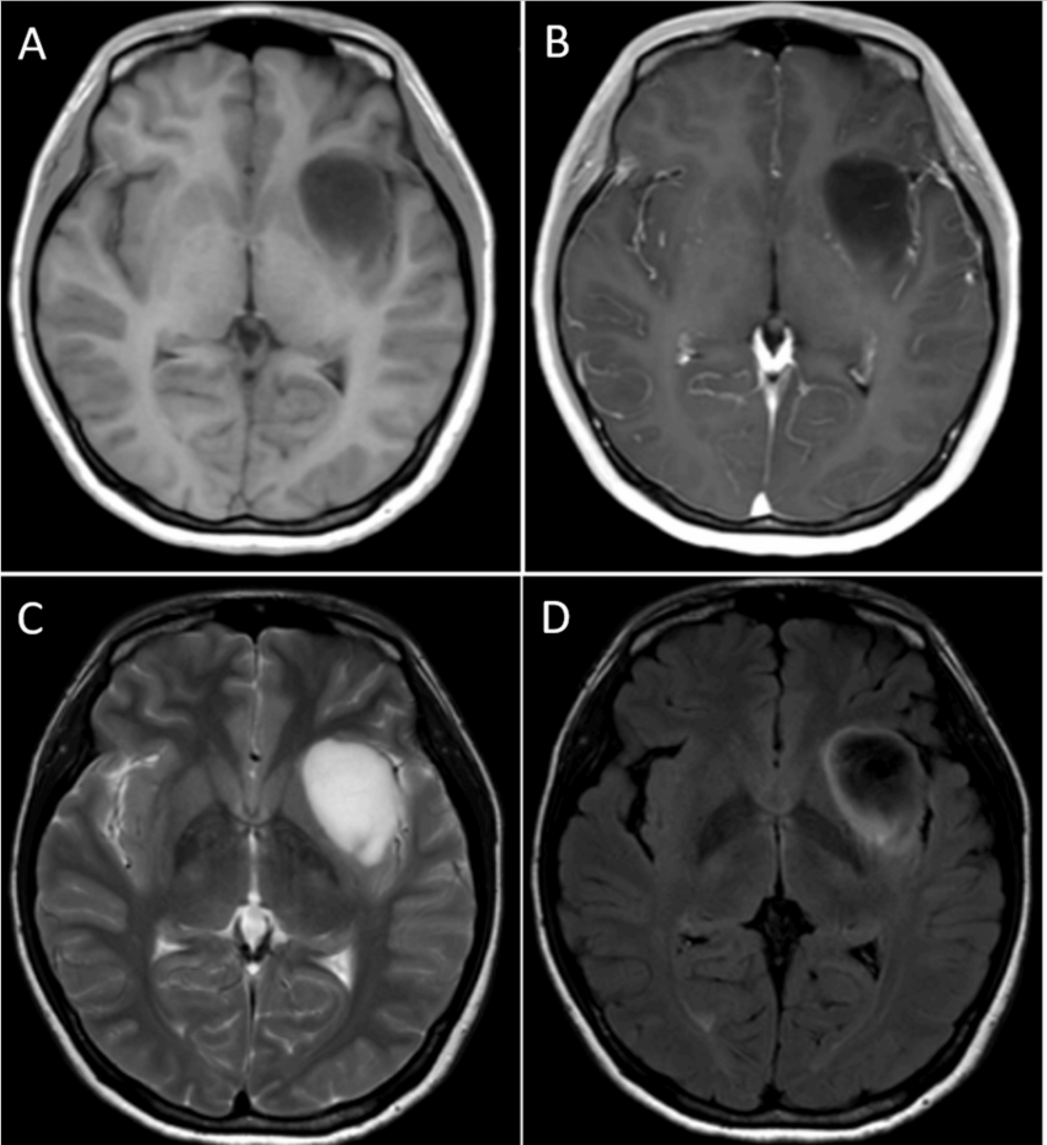
Preoperative MRI demonstrating a left diffuse insular glioma. The T1-weighted image (A) and T1 contrast-enhanced image (B) show an enhancing rim surrounding the lesion. The T2-weighted image (C) and FLAIR image (D) exhibit a T2-FLAIR mismatch sign, suggestive of an IDH-mutant astrocytoma. IDH: isocitrate dehydrogenase; FLAIR: Fluid-Attenuated Inversion Recovery

**Figure 2:**
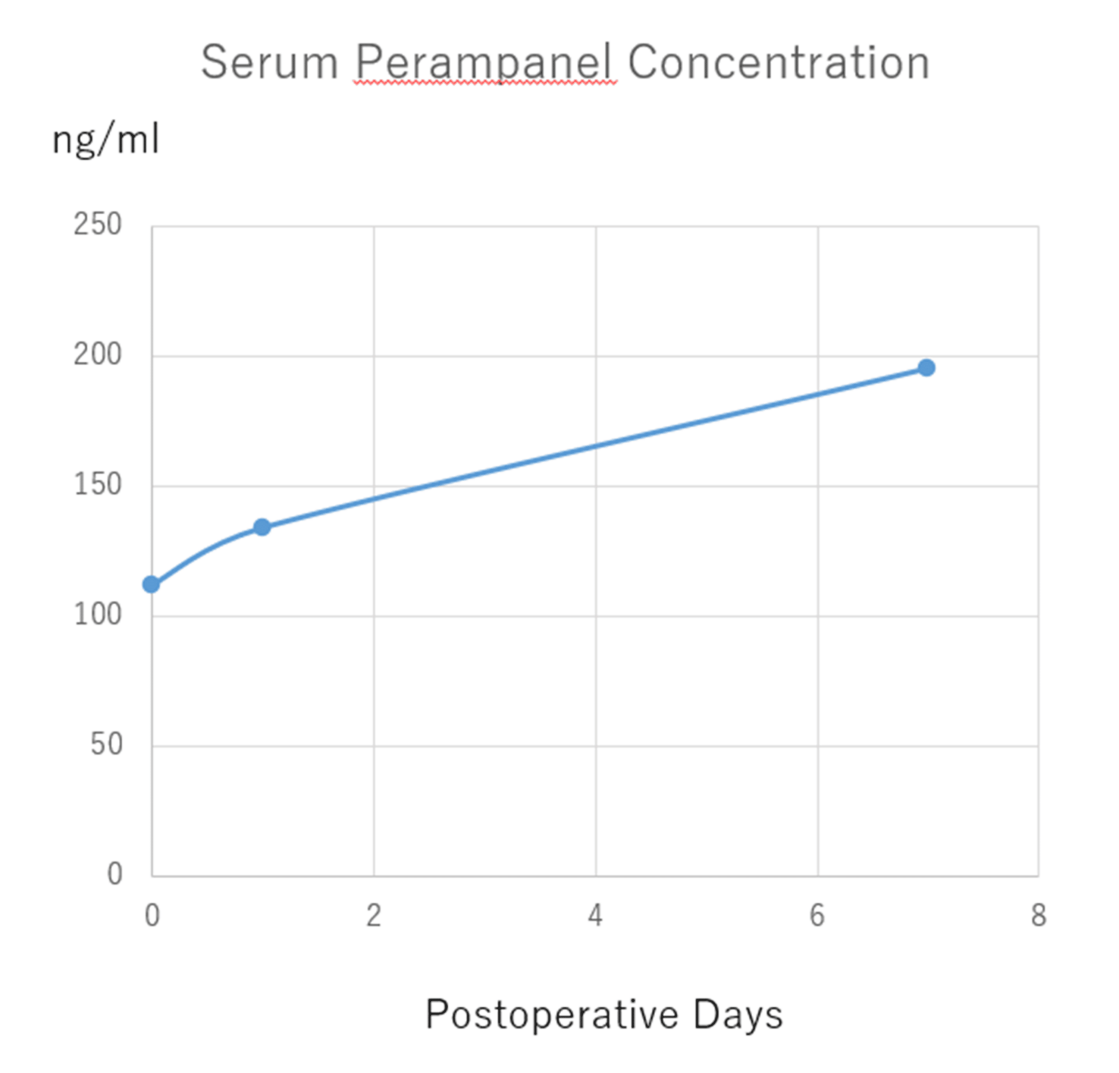
Serum concentrations of perampanel measured on the day of surgery (Day 0) and during the early postoperative days. Levels remained elevated throughout the first postoperative week following a single intraoperative dose.

**Figure 3:**
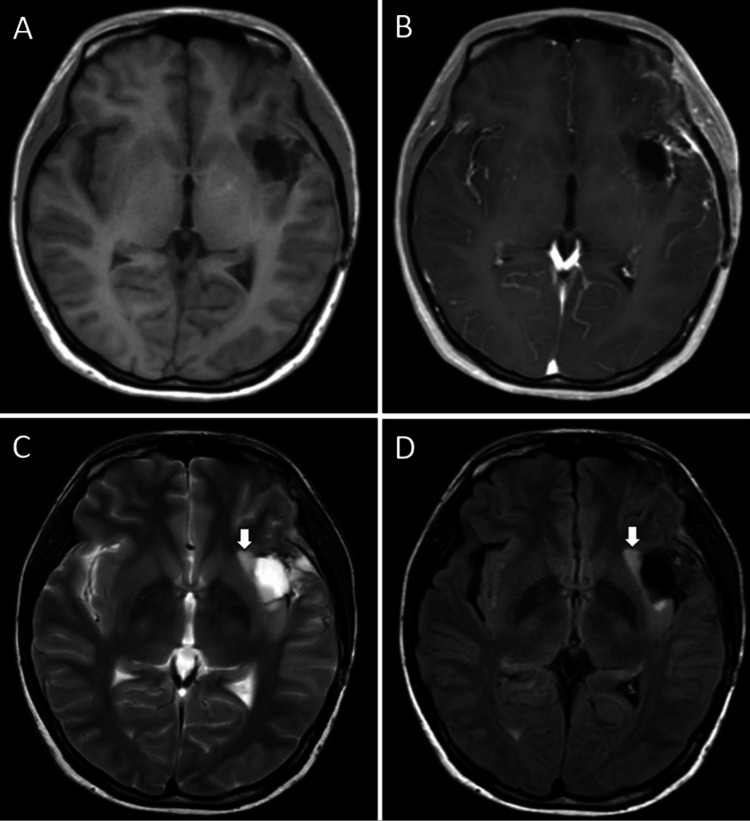
Postoperative and post-chemoradiation MRI demonstrating residual diffuse insular glioma. The non-contrast T1-weighted image (A) and contrast-enhanced T1-weighted image (B) show removal of the previously enhancing rim. The T2-weighted image (C) and FLAIR image (D) demonstrate residual tumor (arrows).

A 35-year-old right-handed female (body weight: 40 kg) presented with a headache. Magnetic resonance imaging (MRI) revealed a left insular diffuse glioma (Figure 1). She had no history of epilepsy and was not on any ASMs. No routine electroencephalogram (EEG) was performed preoperatively. A left awake craniotomy was performed using the asleep-awake-asleep technique for tumor resection. A 6 mg intravenous dose of perampanel (dissolved in 100 mL of normal saline and infused over 60 minutes) was administered at the beginning of surgery in the operating theater. Following standard craniotomy and exposure of the insula during the initial asleep phase, intravenous propofol and remifentanil were paused to awaken the patient. She regained consciousness approximately 3.5 hours after perampanel administration, at which time the serum perampanel concentration was 112 ng/mL. During the awake phase, the patient successfully completed motor and language tasks without difficulty. Cortical and subcortical mapping was performed using a bipolar stimulator, while electrocorticographic monitoring was conducted via subdural electrodes placed over the cortical surface. The awake phase lasted approximately 4.5 hours, during which no clinical or electrical seizures were detected. Continuous intraoperative electrocorticography revealed no evidence of subclinical seizure activity. A subtotal resection was achieved, with the medial portion of the tumor deliberately preserved to maintain the integrity of the inferior fronto-occipital fasciculus. Following resection, the patient was re-anesthetized, and the surgical site was closed. Postoperative arousal was uneventful, with an anesthesia awakening time of 37 minutes, measured from the decision to initiate arousal to the completion of surgery. Serum perampanel concentrations measured on postoperative days 1 and 7 were 134 ng/mL and 195 ng/mL, respectively (Figure 2). The patient demonstrated no immediate motor, sensory, or language deficits following surgery. However, motor aphasia emerged on postoperative day 3. Follow-up MRI revealed no evidence of significant ischemic stroke, hemorrhage, or infection. Postoperative serum analyses were unremarkable. No additional clinical signs suggestive of perampanel-related adverse effects - such as cognitive slowing or somnolence - were observed. A standard 21-electrode scalp EEG, recorded for approximately 30 minutes the following day, showed no epileptiform discharges. Oral lacosamide (100 mg/day) was initiated on postoperative day 8; however, no symptomatic improvement was observed. Gradual recovery of motor aphasia occurred with rehabilitation, leading to complete resolution within approximately one month. Lacosamide was discontinued on postoperative day 40, with no subsequent recurrence of symptoms. Histopathological examination confirmed astrocytoma, IDH-mutant, CNS WHO grade 3. The patient underwent postoperative radiotherapy (60 Gy in 30 fractions) and temozolomide chemotherapy. An MRI performed approximately three months after surgery, following completion of chemoradiation, demonstrated residual diffuse insular glioma (Figure 3). At that time, she remained asymptomatic.

## Discussion

Choices of antiseizure medication (ASMs) for awake craniotomy

Nossek et al. investigated 424 patients undergoing awake craniotomy and reported an overall failure rate of approximately 6%, primarily due to a 4% incidence of inadequate intraoperative communication and a 2% incidence of intraoperative seizures [1]. Notably, a history of seizures and preoperative ASM treatment were associated with increased risk of intraoperative seizure-related failure [1]. Despite the critical need to prevent intraoperative seizures while minimizing adverse effects, there is currently no consensus on which ASMs should be routinely administered during awake craniotomy, particularly in seizure-naïve patients. Historically, phenytoin, valproate, and phenobarbital have been employed to manage post-neurosurgical seizures; however, these older agents are frequently associated with significant side effects and drug-drug interactions. Intravenous levetiracetam has emerged as the most commonly used ASM for seizure prophylaxis in oncologic neurosurgery, reportedly utilized in approximately 80% of cases, followed by phenytoin at 20% [10,11]. Levetiracetam has demonstrated superior efficacy over phenytoin in reducing postoperative seizures following craniotomy for brain tumors, although the overall incidence of adverse drug reactions between the two agents appears comparable [12]. In neurosurgical intensive care settings, the combination of lacosamide and levetiracetam has shown similar effectiveness in seizure prevention compared to phenytoin plus levetiracetam, with a more favorable side effect profile [13]. However, data specifically addressing ASM selection for awake craniotomy remain limited. Importantly, intraoperative administration of phenytoin has been significantly associated with failed awake craniotomy procedures due to adverse effects such as somnolence, delirium, and confusion, which impair patient cooperation [1,12]. Recent evidence suggests that a combination of intraoperative intravenous levetiracetam and preoperative oral perampanel (2 mg daily for two weeks) provides superior seizure prophylaxis compared to levetiracetam monotherapy in patients with glioma undergoing awake craniotomy [4]. Nonetheless, the efficacy of intravenous perampanel alone as a preventive strategy in this context has yet to be established.

Perampanel

Perampanel is a fast-acting and long-lasting ASM. It reaches its peak plasma concentration within 0.5 to 2.5 hours and rapidly exerts its antiseizure effects due to its high permeability across the blood-brain barrier [7, 14]. Its plasma half-life ranges from 52 to 129 hours following a single dose [15], supporting sustained therapeutic efficacy for up to one week [14,15]. Intravenous perampanel reportedly exhibits similar pharmacokinetics to its oral formulation [9]. These unique pharmacokinetic properties may make it particularly useful as a prophylactic ASM both intraoperatively and early postoperatively. We propose that the newly introduced intravenous formulation of perampanel may be a valuable option for seizure prophylaxis in awake craniotomy.

Advantages of intravenous perampanel over oral formulation

The intravenous formulation of perampanel offers several advantages over the oral route, particularly in perioperative settings. Although oral administration is typically scheduled for the morning of surgery, it may be challenging in patients with impaired consciousness, swallowing difficulties, a history of nausea or vomiting, or compromised gastric absorption. Moreover, intraoperative delivery via nasogastric tubes can increase the risk of vomiting or aspiration during awake-asleep-awake procedures. In contrast, intravenous administration generally provides more consistent and predictable plasma concentrations. Given that intravenous perampanel reaches its peak plasma concentration within approximately 0.5 to 2.5 hours and maintains therapeutic efficacy for up to a week following a single dose, we administered it at the beginning of the first asleep phase (i.e., when the patient was positioned on the operative table). We anticipated that its plasma concentration would peak by the end of the asleep phase or the beginning of the awake phase, thereby maximizing seizure prophylaxis while minimizing potential adverse effects. The intravenous formulation thus offers more predictable pharmacokinetics, enhancing clinical reliability.

Dose of intravenous perampanel

The optimal dosage for single intravenous administration of perampanel during awake craniotomy remains undefined. In standard clinical practice, oral perampanel administration typically starts at 2 mg to minimize adverse events. Dizziness and somnolence are the most frequent adverse events after oral administration, occurring in approximately 5-30% and 1-20% of cases, respectively [8]. Less common adverse events include headache, irritability, and nasopharyngitis. In clinical practice, oral doses are gradually increased to 4 or 8 mg/day over several weeks to mitigate these risks [8]. In this case, a single 6 mg intravenous dose of perampanel was administered intraoperatively. A key concern in dose selection was the risk of somnolence during the awake phase, which could compromise the patient’s ability to perform motor and language tasks. Consequently, a higher dose (e.g., 8-12 mg) was avoided due to the potential for adverse effects during the awake phase. Serum concentrations during surgery and the early postoperative period ranged from 110 to 200 ng/mL, which falls below the previously reported therapeutic range of 200-600 ng/mL [16], suggesting that the administered dose may have been subtherapeutic. The 35-year-old female patient remained fully alert and successfully completed all intraoperative tasks over a 4.5-hour awake phase without experiencing seizures or observable adverse effects following the 6 mg dose. The efficacy and optimal dosing of perampanel in the context of awake craniotomy remain uncertain, particularly with respect to patient-specific variables such as age, body weight, and renal or hepatic function. To establish evidence-based dosing protocols-especially those personalized to individual physiological characteristics-further investigation is needed. Ideally, this would involve randomized controlled trials with larger and more diverse patient cohorts.

Prophylactic use of perampanel for early postoperative seizures

Our findings suggest that perampanel may also be potentially effective in preventing early postoperative seizures. These seizures are typically defined as occurring within 1 to 2 weeks following brain surgery [17, 18], with an estimated incidence of approximately 8% among patients undergoing awake craniotomy [19]. Prior studies have demonstrated that levetiracetam and phenytoin can significantly reduce the risk of early seizures - particularly within the first 7 days - compared to no prophylactic treatment [10, 18]. The American Association of Neurological Surgeons advises that decisions regarding ASM administration during the first postoperative week should be made at the discretion of the operating surgeon [18]. In our case, the patient developed motor aphasia during the first postoperative week, which persisted for approximately one month. However, the absence of epileptiform activity on EEG and the lack of clinical change following lacosamide administration suggested that neither early postoperative seizures nor perampanel-related toxicity were likely contributors. Motor aphasia is a well-documented consequence of left-sided anterior insular injury, such as that caused by stroke [20]. Our findings suggest that a single intraoperative dose of perampanel may also offer potential efficacy in reducing the risk of early postoperative seizures. However, the optimal dosing strategy remains undefined and warrants further investigation.

Ethical consideration

The use of intravenous perampanel for seizure prophylaxis in seizure-naïve patients undergoing awake craniotomy represents off-label administration. However, the prophylactic use of off-label ASMs is a well-established clinical practice in neurosurgical settings and frequently contributes to the safe and effective execution of awake craniotomy procedures. In this context, the application of intravenous perampanel is considered ethically justifiable. Institutional review board approval was obtained, and written informed consent was provided by the patient.

## Conclusions

This case represents the first documented use of a single intraoperative intravenous dose of perampanel administered at the beginning of awake glioma surgery in a seizure-naïve patient with the intent to prevent both intraoperative and early postoperative seizures. The rapid onset and sustained serum levels of perampanel suggest potentially favorable pharmacokinetic properties for use in awake craniotomy. However, given that this is a single case report with subtherapeutic serum concentrations, further research is necessary to validate these preliminary observations. In particular, studies involving larger and more diverse patient populations are needed to establish optimal dosing strategies tailored to individual factors such as age, body weight, and organ function.
